# Oral *Candida* Infection in Psoriatic Patients Treated with IL17A Inhibitors: Report of 3 Cases and a Comprehensive Review of the Literature

**DOI:** 10.3390/diagnostics12010003

**Published:** 2021-12-21

**Authors:** Efstathios Pettas, Vasiliki Savva, Vasileios Ionas Theofilou, Maria Georgaki, Nikolaos G. Nikitakis

**Affiliations:** 1 Department of Oral Medicine & Pathology and Hospital Dentistry, School of Dentistry, National and Kapodistrian University of Athens, 11527 Athens, Greece; stathis.pettas12@gmail.com (E.P.); vtheofilou@umaryland.edu (V.I.T.); nnikitakis1@yahoo.com (N.G.N.); 2 School of Dentistry, National and Kapodistrian University of Athens, 11527 Athens, Greece; vickysavva48@gmail.com; 3 Department of Oncology and Diagnostic Sciences, School of Dentistry, University of Maryland, Baltimore, MD 21201, USA

**Keywords:** oral candidiasis, interleukin 17, type 17 immunity, inhibitor, secukinumab, brodalumab, psoriasis

## Abstract

An intact and fully functional immune system plays a crucial role in the prevention of several infectious diseases. Interleukin (IL)17 is significantly involved in oral mucosa immunity against several antigens and microorganisms, including *Candida albicans* (CA). Herein, we present three cases of oral candidiasis (OC) related to the use of an IL17A inhibitor for psoriasis. Three psoriatic individuals presented for evaluation of widespread symptomatic oral lesions temporally correlated with the onset of IL17A inhibitors (secukinumab in two patients and brodalumab in one patient). Clinical examination revealed either partially removable white plaques in an erythematous background (case #1) or diffuse erythematous lesions (cases #2 and 3) involving several areas of the oral mucosa. Cytology smear, accompanied by histopathologic examination in case #1, confirmed the clinical impression of OC in all three cases. All patients received antifungal therapy with satisfactory clinical response. No discontinuation of the antipsoriatic regimen was recommended, but all patients were advised to remain under monitoring for possible OC relapses. During the last few years, new systemic biologic agents targeting IL17 have been used for the management of variable immune-mediated diseases. Few clinical trials and scarce case reports have shown that these medications place individuals at high risk of developing candidiasis. We propose that patients treated with these medications should be at close monitoring for the development of OC and, if it occurs, receive appropriate management.

## 1. Introduction

Interleukin (IL)17, a well-recognized cytokine discovered in 1993 [1], plays a decisive role in immunology, also implicated as a driver of multiple signals in the pathogenesis of several autoimmune and/or autoinflammatory diseases [2]. Research findings support IL17 to be the “conductor” of a pathway that leads to protective immunity [3]. IL17-expressing populations include T helper (_H_) 17 subset of T cells and innate-acting lymphocytes [4]. Although mainly produced by T-lymphocytes, IL17 secretion from B cells and myeloid cells has also been shown but still remains controversial [3]. IL17 family consists of six members (from IL17A to IL17F), of which IL17A and IL17F share the same receptor (IL17RA and IL17RC) in both epithelial and mesenchymal cells [3]. Inflammation or signals derived from pathogen-associated molecular patterns (PAMPs) together with IL1 and IL23 promote the expression of IL17, with RORγt and STAT3 being the main transcription factors implicated in this process [3]. The recruitment of ACT1 (also known as TRAF3IP2) in the IL17R receptor is essential for the beginning of IL17 signaling events [3]. Hereupon, the accumulation of several transcription factors, such as TRAF6 and C/EBP, as well as G-CSF, a proinflammatory cytokine, results in a targeted gene expression leading to the formation of the host’s armamentarium against antigens [3,5].

*Candida albicans* (CA), the species typically blamed for the most common fungal infection in humans, is a target microorganism of IL17-mediated immune responses. CA is a member of healthy human mycobiota, colonizing several areas of the human body, including the oral mucosa [6]. In immunocompetent patients, the fungus exists in harmony with other members of the microbiome [6]. However, dysbiosis of oral microbiota and disruption of this balance may enable the CA burden to expand and cause infection [6]. In the oral cavity, β-glucans exposed by *Candida* hyphae regulate the activation of the Dectin-1/CARD9 pathway, which leads to the production of IL1, IL6, and IL23 by the antigen-presenting populations found in the epithelium [3]. Candidalysin, a peptidase secreted by the hyphal form of CA, damages oral epithelial cells resulting in the secretion of IL1 and IL36 [3]. These signals drive the expression of IL17, which orchestrates several immune events inducing fungicidal β-defensins, as well as neutrophil-activating chemokines CXC and hemopoietin G-CSF [3].

The role of IL17 in the pathogenesis of various immune-medicated disorders, including rheumatoid arthritis, ankylosing spondylitis, chronic inflammatory intestinal diseases, multiple sclerosis, and psoriasis, has been described in the literature [2,7]. Psoriasis is a chronic autoimmune-autoinflammatory disorder characterized by abnormal differentiation and uncontrollable proliferation of epidermal cells. IL17A, IL17C, and IL17F levels have been found to be significantly increased in psoriatic individuals [8], with IL17A being the one most biologically active [9], while a synergistic role of TNF-α has been also suggested [10]. Keratinocytes, endothelial cells, and innate immune cells are the main targets of type 17 immunity [11].

In recent years, new systemic biologic agents targeting IL17 have been approved for the management of psoriatic patients. Secukinumab and ixekizumab, two anti-IL17A monoclonal antibodies, as well as brodalumab, a monoclonal antibody binding to IL17RA and neutralizing IL17 function, are the ones more commonly used [12,13]. Bimekizumab, a more recently manufactured antibody, neutralizes both IL17A and IL17F [12]. Recent findings, as well as knowledge of their mechanistic effects in type 17 immunity, indicate that these medications place patients at high risk of developing candidiasis [14]. Our scope is to present three cases of oral candidiasis (OC) related to IL17 inhibitors administered in psoriatic patients, underlining the need for awareness of this adverse effect and highlighting the appropriate diagnostic work-up and management.

## 2. Case Reports

### 2.1. Case 1

A 22-year-old female presented for evaluation of mildly symptomatic oral lesions, firstly noticed a day before. The patient had been diagnosed with cutaneous psoriasis 7 years ago, for which she was recently treated with one subcutaneous injection of secukinumab (Cosentyx^®^) 300 mg per month; the last injection was performed a week ago. She reported occasional weakness after injection of the aforementioned drug. A complete blood count (CBC), performed 3 months ago, was within normal limits.

**Figure 1:**
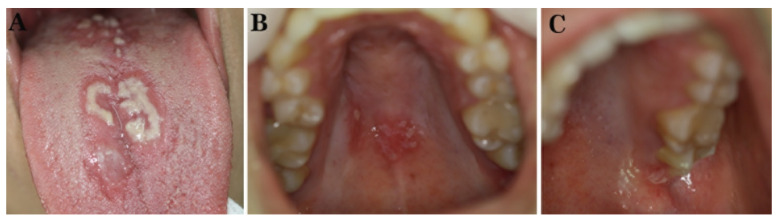
Patient 1. Clinical examination: Partially removable white plaques on erythematous base involving the dorsal tongue (**A**), hard palate (**B**), and left tuberosity (**C**).

**Figure 2:**
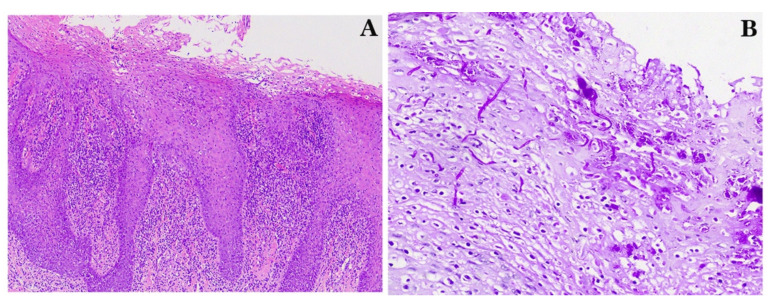
Patient 1. (**A**) Histopathologic examination revealing hyperparakeratosis, neutrophilic microabscesses in the upper layers, thickened spinous layer with elongated rete pegs, and inflammation of the underlying connective tissue with exocytosis (H&E, initial magnification 100×). (**B**) Tubular hyphae of *Candida albicans* are seen embedded in the parakeratin layer (PAS staining, initial magnification 200×).

Clinical examination revealed white plaques on an erythematous base, which were only partially removable, leaving a hemorrhagic surface located on the dorsal tongue (more exuberant) (Figure 1A), hard palate (Figure 1B), and left tuberosity (Figure 1C). To further corroborate a provisional diagnosis of pseudomembranous and chronic hyperplastic candidiasis (CHC) and rule out other entities in the differential diagnosis with a similar clinical appearance, an incisional biopsy, as well as a cytology smear, of the dorsal tongue were performed. Histopathologic examination showed hyperparakeratosis, acanthosis with elongated rete ridges, in addition to mixed, mainly chronic, inflammation of the underlying connective tissue with neutrophilic exocytosis and microabscesses formation in the upper epithelial layers (Figure 2A), while staining with Periodic acid-Schiff (PAS) revealed tubular hyphae of CA in the parakeratin layer (Figure 2B). A cytology smear was also positive for the presence of CA hyphae. On the basis of these microscopic findings, the diagnosis of OC was confirmed. Considering the temporal relationship between the onset of the symptoms and the administration of the last secukinumab injection in the absence of other predisposing factors for OC, an etiologic relationship between secukinumab administration and OC development was considered very likely.

**Figure 3:**
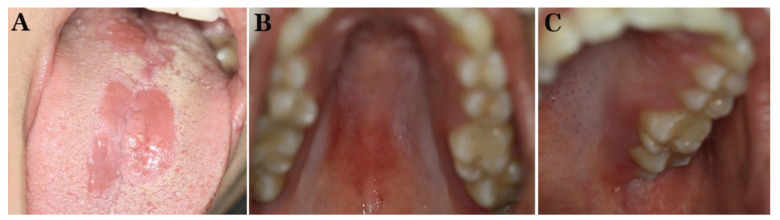
Patient 1. Clinical examination one week after the onset of the antifungal therapy: significant improvement of the lesions on the dorsal tongue (**A**), hard palate (**B**), and left tuberosity (**C**).

The patient was prescribed systemic fluconazole 100 mg qd in combination with miconazole oral gel 20 mg qid and chlorhexidine 0.2% oral rinses bid for 2 weeks. On follow-up after one week, the patient was free of any symptoms, and the clinical examination revealed resolution of the white patches and improvement of the erythematous areas (Figure 3A–C). She was instructed to continue the antifungal therapy for another week; also, she was informed about the possible relationship between the antipsoriatic treatment and the development of OC and was instructed to discuss this side effect with her dermatologist. She was further advised to undergo monitoring with proper oral examination when receiving the injection with prompt intervention in case of OC recurrence. Two weeks after her last appointment, the patient reported a total absence of clinical symptoms as well as complete resolution of the lesions. However, she was subsequently lost to follow-up.

### 2.2. Case 2

A 66-year-old female presented for evaluation of erythematous lesions on her oral mucosa; she also reported burning mouth symptoms, taste dysfunction, and xerostomia. The patient was diagnosed with cutaneous psoriasis 30 years ago and, for the last 3 years, was under treatment with secukinumab (Cosentyx^®^) 150 mg (two subcutaneous injections monthly). Her medical and drug history was otherwise unremarkable, and a CBC performed 5 months ago was within normal limits. Although fluctuating for almost 5 years, the oral symptoms became more intense with the commencement of secukinumab; therefore, a possible relationship with the antipsoriatic medication was considered.

**Figure 4:**
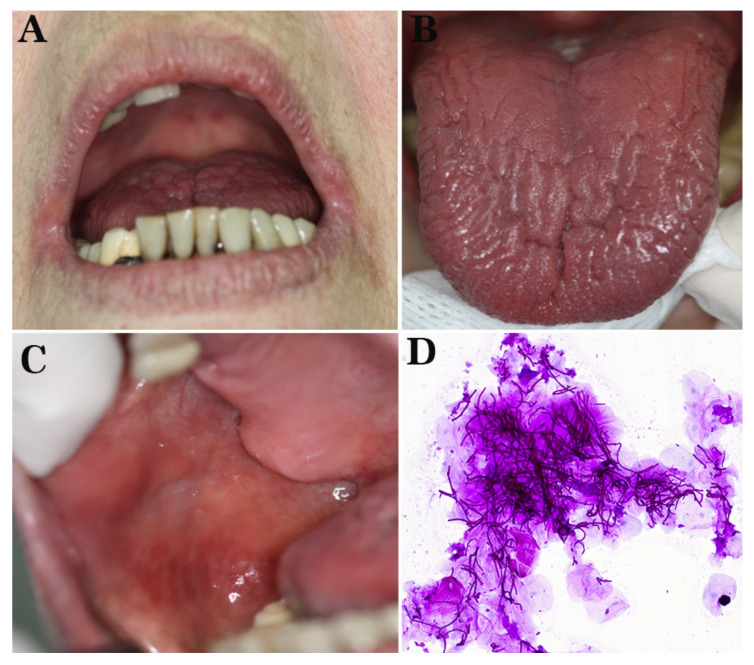
Patient 2. (**A**–**C**): Clinical examination: dryness and erythema in the labial commissures (**A**), erythematous glossy dorsal tongue (**B**), and diffuse erythema in the right buccal mucosa (**C**). (**D**) A cytology smear from the dorsal tongue demonstrating yeasts of *Candida albicans* (PAS staining, initial magnification 200×).

Clinically, dryness and erythema in the labial commissures (Figure 4A), an erythematous glossy dorsal tongue (Figure 4B), diffuse erythema in the buccal mucosae (Figure 4C), as well as erythematous patches on the hard palate were clinically observed, while salivary flow rate was normal. A cytology smear from the dorsal tongue was performed and stained with PAS, showing yeasts of CA (Figure 4D). A final diagnosis of erythematous candidiasis and angular cheilitis was made.

The patient was prescribed miconazole oral gel 20 mg qid and chlorhexidine oral solution 0.20% bid, while a cream of miconazole nitrate 20 mg combined with hydrocortisone 10 mg was also applied qid to the labial commissures for 2 weeks. She was advised to consult with her dermatologist and to remain under monitoring with appropriate oral examinations. Two weeks later, the patient reported significant improvement of the clinical symptoms and was subsequently lost to follow-up.

### 2.3. Case 3

A 50-year-old male presented for evaluation of tongue lesions causing burning symptoms of approximately 10 months duration. Two years ago, the patient was diagnosed with cutaneous psoriasis, for which, until recently, he was receiving two subcutaneous injections brodalumab (Kyntheum^®^) 210 mg per month; the injections were discontinued 3 months ago. His medical history was also significant for tuberculosis, diagnosed two years ago, for which he received isoniazid (3 tablets per day) for 9 months; he clearly reported that the oral symptoms presented several months after the discontinuation of isoniazide, while he was receiving only brodalumab; therefore, a possible relationship of the latter with oral lesions was considered. A recently performed CBC was within normal limits.

**Figure 5:**
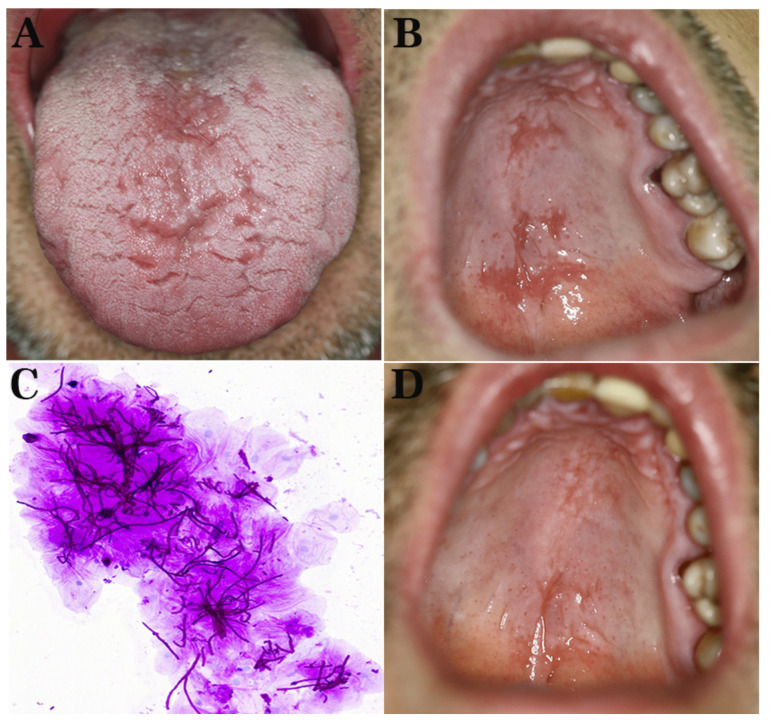
Patient 3. (**A**,**B**): Clinical examination: scattered erythematous areas on the dorsal tongue (**A**) and hard palate (**B**). (**C**) Cytology smear from the dorsal tongue showing yeasts of *Candida albicans* (PAS staining, 200×). (**D**) Clinical examination one week after the commencement of antifungal treatment showing significant remission of the palatal lesions.

Clinically, dryness and erythema in the labial commissures, as well as scattered erythematous areas on the dorsal tongue (Figure 5A) and hard palate (Figure 5B), were noticed. A cytology smear from the oral erythematous lesions was performed and stained with PAS demonstrating yeasts of CA (Figure 5C). A final diagnosis of erythematous candidiasis and angular cheilitis was rendered, and the patient received systemic fluconazole 100 mg qd in combination with miconazole oral gel 20 mg qid and chlorhexidine 0.20% oral solution bid for one week; a cream of miconazole nitrate 20 mg combined with hydrocortisone 10 mg was also applied to labial commissures qid. At 1-week follow-up, the patient was asymptomatic, demonstrating significant remission of the lesions, chiefly the palatal (Figure 5D). He was advised to continue the antifungal treatment for another week and, in case the antipsoriatic regimen was resumed, to remain under monitoring for OC development.

## 3. Discussion

In this paper, we have presented three cases of OC in patients with no other known predisposing factors, apart from the use of anti-IL17A medications for psoriasis, specifically secukinumab (cases #1 and 2) and brodalumab (case #3). The possibility of candidiasis development in patients receiving such medications has been recognized in several clinical trials, but their diagnosis in routine clinical practice with a detailed presentation of their clinicopathologic features and management has been scarce in the literature.

The reason behind candidiasis development in patients receiving anti-IL17A medications is directly related to the aforementioned role of IL17-mediated immune mechanisms in preventing and controlling *Candida* infections. Indeed, several disorders related to inborn or acquired deregulation of IL17 have been associated with a high frequency of candidiasis. The protective role of IL17 against *Candida* is apparent in human primary immune deficiencies; disorders related to mutations in genes involved in the IL17 pathway are characterized by an increased rate of *Candida* colonization [3]. For example, patients with mutations affecting IL23-dependent IL17A/IL17F immunity, as well as individuals with absence of IL17A/F-producing T cells due to loss-of-function mutations in *RORγ* and *RORγt* genes, suffer from chronic mucocutaneous candidiasis (CMC) [15,16], an uncommon disorder characterized by persistent or recurrent *Candida* infections involving the skin, nails, and mucosae [17]. Similarly, deficiency of T_H_17 cells due to mutations in *STAT3* genes, resulting in lack of IL17 production, place individuals with the autosomal dominant hyper-IgE syndrome (Job’s syndrome) at high risk for CMC or oropharyngeal candidiasis [18]. *Candida* infections also thrive in humans with mutations in genes encoding other molecules involved in IL17-mediated immunity, including *TRAF3IP2* (also known as *ACT1*), *IL17F*, *IL17RA*, and *IL17RC* genes [19,20,21,22]. Recently, a case of CMC in a patient diagnosed with a novel mutation in the *TRAF3IP2* gene, encoding for a protein participating in IL17 receptor downstream signaling, was also reported [23]. Another interesting correlation is that susceptibility to OC among HIV seropositive individuals is likely to be attributed to T_H_17 cells decrease [24].

Novel drugs targeting IL17 have been associated with several adverse effects, some of which may manifest or arise in the oral cavity, including lichen planus/lichenoid mucositis [25,26,27], oral ulcerations [28,29], and herpetic infections [28,30,31,32]. As expected, the development of candidiasis is frequently listed among side effects in several clinical trials involving anti-IL17A inhibitors. Regarding location, *Candida* infection related to these neutralizing antibodies may show a predilection for the oral cavity, followed by the vulvovaginal/genital area, skin, esophagus, and oropharynx [33,34,35]. In a comprehensive review of the English language literature on clinical trials using anti-IL17A inhibitors as targeted therapy for psoriasis, psoriatic arthritis, and ankylosing spondylitis, 25 studies [25,30,32,33,34,35,36,37,38,39,40,41,42,43,44,45,46,47,48,49,50,51,52,53,54], specifically including OC evaluation as a potential adverse event, were identified (Table 1). It should be mentioned that a few additional trials did not report OC as an adverse event, without clarifying whether the infection was not observed or not investigated [28,29,31], while two studies were not evaluated due to insufficient data distinguishing the number of oral or genital infections [55,56]. From the 25 clinical studies evaluated, 16 used secukinumab (in doses of 300, 150, or 75 mg) [25,30,32,33,35,36,38,39,40,41,45,46,47,49,51,52]; 4 used brodalumab (in doses of 210, 140, and 70 mg) [37,42,43,54]; 4 used bimekizumab (in dosages ranging from 40 to 560 mg) [44,48,50,53]; and 1 study used ixekizumab (in a dose of 80 mg) [34]. Interestingly, in three clinical trials with secukinumab [25,38,45] and one with bimekizumab [44], none of the patients developed OC infection during the study period.

Regarding secukinumab, the frequency of OC development in various trials (per dosage category) ranged from 0% to 5%; the total number of patients treated in the various trials was 4.350, with 36 (0.83%) developing OC. According to the dosage used (in studies specifying adverse effects per dose), treatment with 300 mg, 150 mg, or 75 mg resulted in OC development in 7 out of 553 (1.26%), 16 out of 2146 (0.75%), and 9 out of 1154 (0.78%), respectively, indicating that higher doses of secukinumab are more likely to result in oral *Candida* infection. In contrast, analyzing the results of a few previous studies comparing different doses of secukinumab treatment, OC appears to be a dose-related adverse event only in some studies [39,55], but not in others [30,32,33,40,46].

In trials using brodalumab, OC occurrence appeared to be higher compared to secukinumab, ranging from 0.9% to 7% in various studies for an overall incidence of 3.8% (16 out of 418 total cases). Even higher percentages of OC were recorded in studies with bimekizumab, affecting 43 out of a total of 669 (6.4%) patients. A single but sizable study of ixekizumab, consisting of 3736 patients, reported OC development in 63 patients (1.7%) [34].

The aforementioned clinical trials provide sufficient evidence that OC is one of the relatively frequent side effects of anti-IL17A medications. However, a direct etiologic relationship on a case-by-case basis is difficult to prove, while, in a few of these studies, other confounding factors may exist, such as the use of medications that could (either directly or indirectly) provoke candidiasis development, such as oral steroids, immunomodulatory agents (e.g., methotrexate), and antidepressants [33,47,53]. Further, correlations with patients’ demographics cannot be made, since almost all clinical trials do not provide the specific demographic characteristics of those who developed OC; however, a study evaluating elderly patients (above 65 years of age) reported that no significant association between the frequency of adverse events, including OC, and their specific age was observed [57]. In relationship to diagnostic methodology, the specific work-up used to diagnose OC is not detailed in most clinical trials, which also do not describe the specific site and clinical appearance of lesions. In terms of severity, the majority of OC cases reported in trials were mild or moderate [41,50], even resolving without any antifungal therapy, however without specifying the method of severity assessment; few studies reported severe cases [33,34] that even led to discontinuation of the medication in a minority of patients [47], which raises concerns whether the infection was indeed limited to the oral cavity or extending to the oropharynx. For example, Ritchlin et al. [53] reported two cases with mild or moderate oral *Candida* infection that required short interruption of treatment and systemic antifungal therapy. Nonetheless, most studies lacked details about therapeutic interventions, such as administration, type, and duration of antifungal therapy and the need or not for discontinuation of the anti-IL17A medication, as well as follow-up information, including the possible reappearance of the infection with discontinuation of the antifungal medication or restart of the biologic agent.

Besides clinical trials, the number of case series and reports specifically addressing the development of OC in patients receiving anti-IL17A regimens remains very limited [27,58,59,60,61,62]. In this regard, our three cases represent uncommonly reported examples of microscopically proven OC with the characteristic clinical appearance in patients without any other predisposing factor besides anti-IL17A treatment, while case #3 is the first well-documented OC related to brodalumab published to this date. In a detailed case series of 16 psoriatic patients [59], two of which had predisposing factors for candidiasis (the one was diabetic and the other had recently completed a 3-month treatment with steroids), none of them developed oral (or other mucosal or cutaneous) *Candida* infection, as assessed by swabs and culture, during the 12-month therapy periods with subcutaneous injections of secukinumab 300 mg monthly. Recently, a case of microscopically confirmed OC in a 52-year-old female patient with psoriasis treated with secukinumab (300 mg monthly) was reported by Farah [60]. Specifically, a thick white plaque on the dorsal tongue, consistent with CHC, was noticed and successfully treated with oral fluconazole 50 mg per day for 3 weeks; maintenance of the targeted immunotherapy was decided, and no recurrences were noticed. Interestingly, in addition to CHC, the patient also presented with asymptomatic oral lichenoid lesions (not biopsy-proven), which were also attributed to secukinumab, although a clear correlation with the onset of the medication could not be supported. Picciani et al. [58] reported a similar case of a 50-year-old psoriatic female patient on secukinumab (300 mg monthly), who also presented OC involving the tongue in the form of erythema and atrophy of the dorsal surface and non-removable white plaques on the lateral border; the lesions developed 60 days after commencement of the antipsoriatic medication. A suitable response was achieved with topical application of miconazole oral gel 20 mg qid for 30 days, and the patient was recommended to discontinue the antipsoriatic treatment. After complete remission of OC, the dose of secukinumab was reduced at 150 mg per month without recurrences during a 7-month follow-up period; this observation is in agreement with the aforementioned higher incidence of OC in higher (300 mg) compared to lower doses of secukinumab in clinical trials. Komori et al. [61] reported a 74-year-old female patient suffering from psoriasis vulgaris and rheumatoid arthritis, who, five months after commencement of secukinumab treatment, developed a painful whitish plaque on her buccal mucosa, microscopically diagnosed as “oral lichen planus with candidiasis”, the later confirmed by PAS staining. Management included secukinumab discontinuation and administration of oral antifungal therapy in the form of oral amphotericin B syrup treatment for two months, which resulted in complete remission of the lesion without subsequent relapses. Similarly, Capusan et al. [27] described a case of a 45-year-old male with psoriasis, who, eight months after the onset of secukinumab, developed oral lichenoid lesions along with OC, confirmed by microscopic examination and culture; clinically, painful ulcerative and white lesions were noticed on the lateral borders and the dorsum of the tongue. Treatment with oral itraconazole and intralesional corticosteroids resulted only in partial remission of the lesions; only after discontinuation of the antipsoriatic treatment for 8 weeks was complete remission observed. Subsequent antipsoriatic treatment with ixekizumab caused a relapse of the OC (without concomitant lichenoid lesions) within a month, which was successfully managed with fluconazole; no further recurrences occurred despite the continuation of the ixekizumab for the next 9 months. Finally, Moldenhauer et al. [62] reported a 59-year-old male patient with psoriasis and psoriatic arthritis who developed candidal balanoposthitis, angular cheilitis, and OC (in the form of only partially removable pseudomembranous patches), in addition to labial and tongue swelling, while on secukinumab treatment (150 mg every 4 weeks) for the last 8 weeks. Notwithstanding, a clear relationship between *Candida* infection and secukinumab could not be safely claimed, as the patient was also under treatment with empagliflozin (SGLT-2 inhibitor) for diabetes type 2, another medication commonly associated with genital *Candida* infections mainly in women (vulvovaginal) [63]. The patient was recommended to discontinue both secukinumab and empagliflozin and received antifungal treatment with fluconazole (200 mg/day per os for 2 weeks), resulting in rapid resolution of the fungal infection; in addition, secukinumab was replaced by ustekinumab (an anti-IL12/IL23 antibody).

Overall, it appears that the majority of aforementioned OC cases, including ours, affected the tongue, followed by the buccal and palatal mucosa and the commissures. In particular, a clinical presentation of CHC, i.e., a non-detachable (or only partially removable) white plaque, appears to be common [58,60], similar to our case #1, while a co-existence of oral lichenoid lesions is also possible [27,60,61]. This propensity for relatively unusual or atypical clinical features, compared to the more typical pseudomembranous or erythematous OC, necessitates ruling out other conditions with clinical similarities. Although OC can usually be diagnosed through the evaluation of a patient’s medical history and symptoms in combination with clinical examination, equivocal cases should be confirmed through detection of *Candida* presence [64,65]. Even though culture can directly identify the organism and also assess its sensitivity to antifungal therapy, this technique may not be readily available in routine clinical practice [64]. The microscopic detection of the hyphae and yeasts could be made in cytologic smears or tissue biopsy samples, commonly stained with PAS [64,65]. A biopsy is mostly recommended for CHC, especially to rule out epithelial dysplasia [64], similar to our case #1.

Regarding management options for OC, the literature supports that topical antifungals are the first-line choice for mild oral *Candida* infections in immunocompetent patients [66]. The most frequently used drugs are nystatin, clotrimazole, and miconazole, commonly prescribed for 1–2 weeks [66,67]. Widespread moderate-to-severe infections not responding to topical medications, as well as immunosuppression, are the most common indications for systemic therapy [66,67]. In such cases, oral fluoconazole 100–200 mg per day for 1–2 weeks seems to be the drug of choice, while itraconazole, voriconazole, and posaconazole may be effective alternative choices [66,67]. Dosage should be individualized depending on the patient’s profile, as well as the severity and type of OC. Regardless of the antifungal regimen, the control of any additional underlying predisposing factor (such as anemia, diabetes mellitus, and immunosuppression) as well as a suitable level of oral hygiene, in addition to the supplementary use of antimicrobial agents, could also contribute to the control of the infection [66,67]. In the previously published OC cases associated with anti-IL17A inhibitors described above, various combinations of topical and/or systemic antifungal treatment were used with overall successful results; similarly, in all three of our cases, a short regimen was sufficient to induce remission within one to two weeks.

In relation to the possible need for drug discontinuation, it seems that OC does not necessarily constitute a reason to discontinue the anti-IL17A inhibitors, as in the case reported by Farah [60]. However, in the other four reported cases [27,58,61,62], discontinuation of the treatment was elected, followed by adjustment of the dosage [58] or replacement of the medication with other of the same [27] or other class [62].

Regarding OC prevention for patients under IL17A inhibitors, the use of lower dosages, especially for secukinumab, may play an important role. Further, these patients should be under close follow-up from both dermatologists and oral medicine specialists in order to detect OC at an early stage, avoiding disruption in the therapeutic regimen with its potentially negative sequelae on the condition under treatment. Prophylactic antifungal therapy could be considered for patients receiving high doses for a long-term period, especially for individuals with other predisposing factors for OC, such as medication-induced immunosuppression. Literature has proven the fungistatic role of all-trans retinoic acid (ATRA), an active metabolite of vitamin A [68]. Noticeably, psoriatic individuals seem to be more susceptible to vitamin A deficiency [69], which could possibly explain the higher frequency of *Candida* infections they commonly face [70]. It was recently suggested that serology testing for vitamin A or ATRA, if possible, could be included in the daily routine evaluation of patients under treatment with anti-IL17A monoclonal antibodies [71].

## 4. Conclusions

Recognizing the role of IL17 in several autoimmune and/or autoinflammatory disorders, including psoriasis, has prompted the successful use of IL17A neutralizing antibodies in their management. However, such medications place these patients at higher risk of OC development since IL17-mediated immune responses serve as essential protectors against *Candida* colonization. Indeed, clinical trials of secukinumab, brodalumab, and ixekizumab have shown an overall OC development incidence of 0.83%, 3.80%, and 1.7%, respectively. Considering that OC may present with a heterogeneous clinical appearance, sometimes masquerading as other oral disease entities, the clinician should be aware of this potential side effect. Patients treated with such inhibitors should be at close monitoring and receive appropriate management if OC develops. As such infections are commonly mild to moderate in severity, biologic agents do not need to be altered or interrupted in the majority of cases.

## Figures and Tables

**Table 1:** Summary of clinical trials of IL17 inhibitor treatment for psoriasis, psoriatic arthritis, ankylosing spondylitis, and rheumatoid arthritis, specifically reporting oral candidiasis development as an adverse effect.

Author	Disease	Treatment Regimen	Frequency of Administration	Duration of Trial	# of Patients (per Treatment Group)	Dosage (per Treatment Group)	% and # of Patients with OC per Treatment Group
Papp et al., 2014 [37]	Psoriasis	Brodalumab	Every 2 weeks	120 weeks	148	210 mg	3.38% (5/148)
Paul et al., 2014 [38]	Psoriasis	Secukinumab	1/weekly to week 4, then every 4 weeks	12 weeks	60 61	300 mg 150 mg	0% (0/60) 0% (0/61)
Blauvelt et al., 2015 [39]	Psoriasis	Secukinumab	1/weekly for the 1st month and then week 8	12 weeks	59 59	300 mg 150 mg	1.79% (1/59) 0% (0/59)
Baeten et al., 2015 [40]	Ankylosing Spondylitis	Secukinumab	Every 2 weeks for the 1st month and then every 4 weeks	52 weeks	276 285	150 mg 75 mg	0.36% (1/276) 0.7% (2/285)
Mease et al., 2015 [33]	Psoriatic Arthritis	Secukinumab	Every 4 weeks	52 weeks	295 292	150 mg 75 mg	1.36% (4/295) 1.37% (4/292)
McInnes et al., 2015 [41]	Psoriatic Arthritis	Secukinumab	1/weekly for the 1st month and then every 4 weeks	52 weeks	133 127 75	300 mg 150 mg 75 mg	1.5% (2/133) 2.36% (3/127) 1.33% (1/75)
Nakagawa et al., 2016 [42]	Psoriasis/ Psoriatic Arthritis	Brodalumab	1/weekly for the 1st month and then every 2 weeks until week 10	12 weeks	37 37 37	210 mg 140 mg 70 mg	0.9% (1/111)
Yamasaki et al., 2016 [43]	Psoriasis/ Psoriatic Erythroderma	Brodalumab	1/weekly for the 1st 3 weeks and then every 2 weeks until week 52	52 weeks	30	140 mg	3.33% (1/30)
Gordon et al., 2016 [34]	Psoriasis	Ixekizumab	Every 2 or 4 weeks	60 weeks	3736	80 mg	1.7% (63/3736)
Braun et al., 2017 [30]	Ankylosing Spondylitis	Secukinumab	Every 4 weeks	104 weeks	181 179	150 mg 75 mg	0.55% (1/181) 0.56% (1/179)
Margo-Ortega et al., 2017 [32]	Ankylosing Spondylitis	Secukinumab	1/weekly for the 1st 3 weeks and then every 4 weeks	104 weeks	106 105	150 mg 75 mg	0.94% (1/106) 0.95% (1/105)
Glatt et al., 2017 [44]	Psoriatic Arthritis	Bimekizumab	Baseline, week 3, 6	20 weeks	20 6 6 6	240/160/160 mg 80/40/40 mg 160/80/80 mg 560/320/320 mg	0% (0/20) 0% (0/6) 0% (0/6) 0% (0/6)
Blanco et al., 2017 [45]	Rheumatoid Arthritis	Secukinumab	Every 2 weeks for the 1st month and then every 4 weeks	52 weeks	215 218	150 mg 75 mg	0% (0/215) 0% (0/218)
Pavelka et al., 2017 [46]	Ankylosing Spondylitis	Secukinumab	Every 2 weeks for the 1st month and then every 4 weeks	52 weeks	113 110	300 mg 150 mg	0.88% (1/113) 0.9% (1/110)
Kivitz et al., 2018 [47]	Ankylosing Spondylitis	Secukinumab	1/weekly for the 1st month and then every 4 weeks	104 weeks	116 117	150 mg 150 mg (no load)	1.73% (2/116) 0.85% (1/117)
Bissonnette et al., 2018 [35]	Psoriasis	Secukinumab	Every 4 weeks	2 or 4 years	168	300 mg	1.19% (2/168)
Papp et al., 2018 [48]	Psoriasis	Bimekizumab	Every 4 weeks until week 8	12 weeks	43 43 40 43 39	480 mg 320 mg 320/160 mg 160 mg 64 mg	0% (0/43) 7% (3/43) 2.5% (1/40) 0% (0/43) 0% (0/39)
Baraliakos et al., 2019 [49]	Ankylosing Spondylitis	Secukinumab	Every 2 weeks for the 1st month and then every 4 weeks	260 weeks	210 64	150 mg 75 mg	1.1% (3/274)
Thaci et al., 2019 [36]	Psoriasis	Secukinumab	Every 4 weeks until week 36	48 weeks	20	300 mg	5% (1/20)
Kishimoto et al., 2019 [25]	Ankylosing Spondylitis	Secukinumab	1/weekly for the 1st month and then every 4 weeks	52 weeks	30	150 mg	0% (0/30)
Blauvelt et al., 2020 [50]	Psoriasis	Bimekizumab	Every 4 weeks	48 weeks	91 111 15	320 mg 160 mg 64 mg	16.5% (15/91) 11.7% (13/111) 6.7% (1/15)
Huang et al., 2020 [51]	Ankylosing Spondylitis	Secukinumab	1/weekly for the 1st month and then every 4 weeks until week 48	52 weeks	453	150 mg	0.44% (2/453)
Pavelka et al., 2020 [52]	Ankylosing Spondylitis	Secukinumab	Every 2 weeks for the 1st month and then every 4 weeks	156 weeks	113 110	300 mg 150 mg	0.45% (1/223)
Ritchlin et al., 2020 [53]	Psoriatic Arthritis	Bimekizumab	Every 4 weeks	48 weeks	80 126	320 mg 160 mg	5% (4/80) 4.8% (6/126)
Yamaguchi et al., 2020 [54]	Psoriasis	Brodalumab	Every 2 or 4 weeks	108 weeks	129	210 mg 140 mg	7% (9/129)

## Data Availability

Department of Oral Medicine & Pathology and Hospital Dentistry, School of Dentistry, National and Kapodistrian University of Athens, Greece.

## Abbreviations

IL: interleukin; CA: *Candida albicans*; OC: oral candidiasis; _H_: helper; PAMPs: pathogen-associated molecular patterns; CBC: complete blood count; CHC: chronic hyperplastic candidiasis; PAS: Periodic acid-Schiff; CMC: chronic mucocutaneous candidiasis; ATRA: all-trans retinoic acid.
